# HLA-B*57 and B*58 Associate with Predictors of Reservoir Size in an Acutely Treated HIV Cohort

**DOI:** 10.1089/aid.2022.0082

**Published:** 2023-03-03

**Authors:** Shida Shangguan, Philip K. Ehrenberg, Aviva Geretz, Lauryn Butler, Suteeraporn Pinyakorn, Somchai Sriplienchan, Carlo Sacdalan, Nitiya Chomchey, Nittaya Phanuphak, Sodsai Tovanabutra, Sandhya Vasan, Denise Hsu, Rasmi Thomas

**Affiliations:** ^1^ U.S. Military HIV Research Program, Walter Reed Army Institute of Research, Silver Spring, Maryland, USA.; ^2^ Henry M. Jackson Foundation for the Advancement of Military Medicine, Inc., Bethesda, Maryland, USA.; ^3^ SEARCH, Institute of HIV Research and Innovation, Bangkok, Thailand.

**Keywords:** HLA, supertypes, acute HIV infection, reservoir size, next-generation sequencing

## Abstract

Much has been learnt about the role of human leukocyte antigen (HLA) alleles during natural infection of HIV-1, but far less is known about their role in people living with HIV (PLWH) on suppressive antiretroviral therapy (ART). In this study we used variable selection to identify predictors of HIV reservoir size, as measured by total HIV DNA in 192 participants in an acute HIV infection (AHI) cohort. Baseline clinical data including pre-ART CD4 T cell counts and plasma viral load (VL) were available from all participants along with longitudinal measurements after ART initiation during AHI. Time to VL suppression, time to CD4 reconstitution, and pre-ART viremia were the strongest predictors of undetectable total HIV DNA at 24 weeks after ART initiation. We next performed HLA typing in 526 participants from the same cohort and investigated associations with the three predictors of reservoir size. HLA-B*57 and B*58 both associated significantly with time to VL suppression, which was one of the predictors of the size of the HIV reservoir. These findings are significant in PLWH and have to be considered in the context of therapeutic intervention when conducting analytic treatment interruption studies as participants with these alleles could impact clinical findings given the small sizes of these studies.

## Introduction

Human leukocyte antigen (HLA) associations with HIV disease progression are well established based on several candidate and genome-wide studies.^1^ However, the successful implementation of antiretroviral therapy (ART) has now led to contemporaneous cohorts of people living with HIV (PLWH). There has been a paucity of HLA association studies in cohorts of PLWH on ART, although assessments of specific HLA alleles previously known to impact HIV outcomes in natural infection cohorts of European ancestry have been described.^2,3^ Similar studies in non-European populations will be needed to assess the effect of HLA in the context of analytic treatment interruption studies to establish if specific therapeutic interventions can prevent viral rebound and eradicate HIV.

Recently in Thailand, a therapeutic vaccine regimen was associated with a significant delay in time to viral rebound after ART interruption, but only after excluding the data from a post-treatment controller with HLA-B*57 in the placebo arm.^4^ Given such associations, it is necessary to identify HLA alleles that might impact viral control or seeding of the reservoir and be a confounder during clinical trials. In this study we investigated total cell-associated HIV DNA levels as a measure of the reservoir, which includes both replication competent and defective viruses, and we also attempt to define HIV outcomes in the context of cohorts treated during acute HIV infection (AHI).

## Methods

Study participants were from the RV254 study,^5^ which enrolled participants with AHI (Fiebig stages 1–5) in Thailand and explored the impact of early ART on immune responses and HIV disease progression. Participants were followed prospectively to gather longitudinal data, including HIV viral load (VL) and CD4 counts at baseline (2–3 days after diagnosis), before and following ART initiation. We selected all available participants from this study for HLA analyses (*N* = 526). All participants from the human studies provided informed consent, and the use of samples for research was approved by institutional review boards in Thailand and the United States.

High-resolution typing of HLA class I loci was performed on all selected participants using next-generation sequencing as described previously.^6^ HLA alleles with frequencies >5% were selected at the one to two field level for further prespecified analyses. Specific alleles of interest with lower frequencies that were previously associated with disease progression were also tested. Reservoir size determined from total HIV DNA levels measured by quantitative PCR was available for a subset of samples with HLA genotyping (*N* = 192).^7^

Several approaches were used to identify HIV outcomes that were predictors of reservoir size. The Mann–Whitney *U* test was used to compare week 0 HIV DNA measurements in 97 individuals assigned to one of the two groups based on reservoir size at week 24. Stepwise regression was used to select significant features that were predictors of week 24 HIV DNA levels (*N* = 192), which was treated as a binary outcome based on the median. Features included in the model were baseline CD4 counts, plasma VL copies/mL, time to CD4 reconstitution (CD4 counts >500), and time to VL suppression (VL <50 copies/mL). The baseline features and the time variables were treated as continuous and categorical variables, respectively, to avoid multicollinearity. Akaike Information Criterion (AIC) was used to identify the optimal variables association with HIV DNA levels at week 24 after ART initiation. Logistic or linear regression was performed to evaluate the association of HLA alleles with significant features in 526 participants. All baseline measurements were log_10_-transformed. Assessment of model diagnostics showed that the assumptions of the linear models were reasonable. Age, sex, and Fiebig stage were adjusted in all models. ART regimen and baseline variables were included additionally for specific models.

A two-sided *p* < 0.05 and *q* < 0.05 was considered significant. All descriptive and inferential statistical analyses were performed using R 4.1.0 (or later) and GraphPad Prism 8 software.

## Results and Discussion

**FIG. 1.**
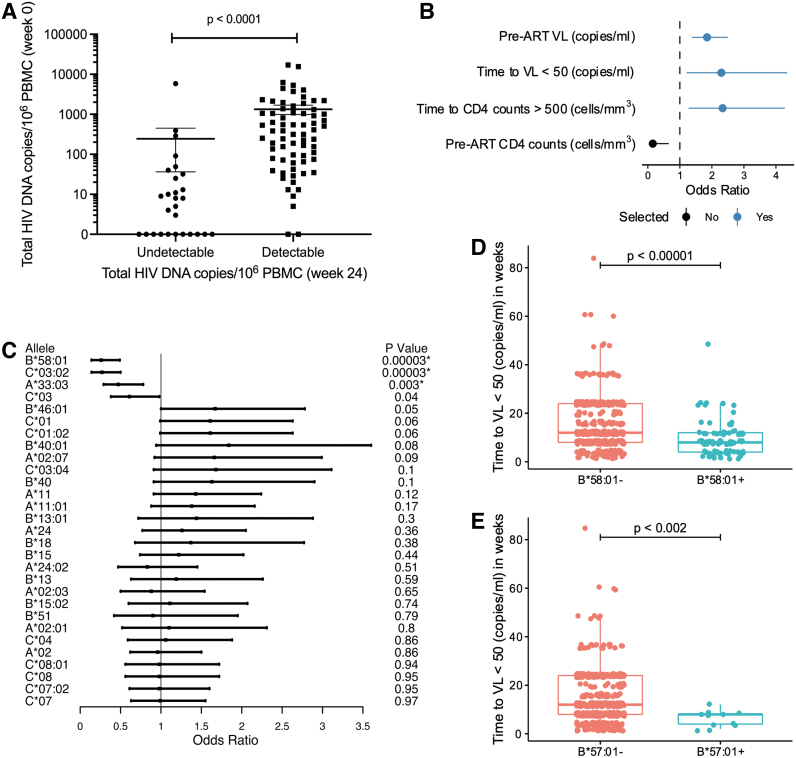
Associations of clinical outcomes with HIV reservoir size after ART initiation. **(A)** Week 0 HIV DNA measurements before therapy are significantly different between groups based on the reservoir size measured from matched samples at week 24 (*n* = 97) after ART initiation. Statistical significance was determined by the Mann–Whitney *U* test. **(B)** Feature selection analyses identified three clinical outcomes that predicted week 24 HIV DNA measurements including baseline VL, time to VL suppression, and time to CD4 reconstitution (*n* = 192). **(C)** Significant association of HLA alleles (frequency >5%) with time to VL suppression (*N* = 526). **(D)** Significance of HLA-B*58 and **(E)** HLA-B*57 with time to VL suppression in an early treated HIV cohort (*N* = 526). *q < 0.05. ART, antiretroviral therapy; HLA, human leukocyte antigen; PBMC, peripheral blood mononuclear cell; VL, viral load.

As HLA variations that influence the reservoir size or reactivation during ART have not been well characterized and may benefit HIV cure research, we investigated HLA associations with HIV outcomes in the RV254 cohort of people who initiated ART during AHI.^5^ Motivated by the scarcity of reservoir measurements in large cohorts, we sought to identify proxies of reservoir size. We performed a variable selection to identify clinical outcomes that most predicted HIV reservoir size in the acutely treated cohort consisting of 526 PLWH. First, baseline and longitudinal HIV DNA levels measurements before ART initiation were analyzed among a subset of 97 participants. We observed in the RV254 cohort that HIV DNA levels measured before initiation (week 0) were significantly different when categorized by HIV reservoir size 24 weeks after starting therapy (Fig. 1A). Therefore, we concluded that pre-ART baseline HIV DNA levels are a proxy of HIV reservoir size (HIV DNA on ART).

This observation prompted further examination of the relationship between other pre- and post-ART initiation clinical measures that could predict reservoir size. These included two pre-ART measures (VL and CD4 count), as well as two measures associated with clinical improvement post-ART initiation (time to achieve normal CD4 counts and time to suppress VL). Pre-ART HIV VL, time to viral suppression, and time to CD4 reconstitution were selected to be the most relevant factors associated with the reservoir size outcome based on feature selection analyses (Fig. 1B). Although higher CD4 counts were significantly associated with lower HIV DNA levels (*p* = 0.01), it was not selected as a relevant feature because it did not improve the overall model in terms of AIC. This was surprising given that we recently showed that HLA-B*46, the most frequent allele in Thailand, was associated with lower CD4 counts and increased disease progression in the same cohort.^8^

We next investigated HLA allele associations with the selected variables in 526 PLWH who began ART treatment during AHI. Of the 29 HLA alleles in the prespecified analysis, A*33:03, B*58:01, and C*03:02 showed a significant correlation with rapid VL suppression (Fig. 1C and Supplementary Table S1). All three alleles are known to be present on the same haplotype and are in strong linkage disequilibrium.^9^ Previous work has shown an association between B*58 and VL before ART in other global populations,^10^ but here we show a strong association with time to decay of viremia after ART initiation (Fig. 1D).

We observed no HLA alleles significantly associated with pre-ART VL (Supplementary Table S2) and time to CD4 reconstitution (Supplementary Table S3). Reasons for not observing HLA associations with pre-ART VL unlike other natural history cohorts could be varied, including timing of HIV infection (acute vs. chronic), VL outcome (single vs. multiple longitudinal measurements), and geographic differences in allele frequencies (Thai vs. European/African).

In addition to our prespecified HLA analysis, for the outcome with significant association, that is, time to VL suppression, we examined low-frequency HLA alleles such as B*57, and B*35 (px) in Thailand, that have previously been shown to be associated with HIV VL and disease progression in other world populations.^11^ We observed that B*57 significantly impacted HIV outcomes among AHI participants from the RV254 cohort (OR = 0.07, *p* = 0.02), and was associated with a faster rate of VL suppression after starting ART (Fig. 1E and Supplementary Table S4). Because differences in ART regimen can influence time to VL suppression (Supplementary Fig. S1), all related associations were also corrected for the regimen used.

In this study we also investigated the association of HLA alleles with HIV outcomes among individuals on ART. Although we did not observe significant associations between HLA alleles and one pre-ART measure of VL, alleles B*57:01 and B*58:01 were associated with reduced time to VL suppression after ART initiation. B*57 has been previously implicated in viral control among treatment-naive cohorts from other world populations and has very recently been identified as a contributing host factor with post-treatment viral control after ART interruption.^4,12^ It is not surprising that alleles associating with viral control before therapy could also influence suppression of the virus after ART initiation.

Both alleles belong to the same B58 supertype and present an immunodominant epitope that significantly compromises viral fitness,^13,14^ and so their underlying mechanisms of viral control might be the same. B*57 and B*58, which are known to have some of the strongest associations with control of viremia during natural infection, are among the alleles we found to associate with events that occur in treated AHI. Both are associated with time to VL suppression, a predictor of HIV DNA levels. Because HIV DNA was measured only in a smaller subset of samples with HLA typing (*N* = 192), we only observed a trend toward significance in HIV DNA levels comparing people with and without B58 supertypes and further studies are warranted (Supplementary Fig. S2A–C). We also acknowledge that total HIV DNA levels measurements may not be reflective of the replication competent reservoir, but such assays are currently unable to target the circulating recombinant CRF01_AE strain.^15^

In this study we used a unique cohort and analysis methods to identify HLA alleles that associate with predictors of total HIV DNA. We present time to VL suppression as a significant predictor of HIV DNA levels. This clinical measurement can help identify alleles that might be involved in HIV control even after ART initiation. These alleles, in turn, may serve as the basis for prescreening cohorts as is performed currently in non-human primates, and may be important factors to consider in the study design and statistical analyses plan of future therapeutic intervention studies. Finally, demonstrating that host genetic effects on HIV can be detected using outcomes such as measurements before ART initiation and time to VL suppression or CD4 reconstitution in treated cohorts suggests that features of current treated cohorts, such as little variation in timing and efficacy of ART, support the possibility of host genetic analyses to improve understanding of contemporaneous infection and its effect on the latent reservoir.

## Supplementary Material

Supplemental data

Supplemental data

Supplemental data

Supplemental data

Supplemental data

Supplemental data
