# Supplementary material for: HLA-B*57 and B*58 Associate with Predictors of Reservoir Size in an Acutely Treated HIV Cohort
Source: AIDS Res Hum Retroviruses. 2023 Mar 3;39(3):114–8. doi: 10.1089/aid.2022.0082 (PMC9986004; doi:10.1089/aid.2022.0082)
Supplement: Supplemental data [file Suppl_TableS1.pdf]

Supplementary Table 1. Association of HLA alleles with time to VL suppression after ART initiation (N = 526)

HLA Allele	N (%)	OR	95% CI	P value (q value*)
A*02	253 (48)	0.96	(0.62, 1.5)	0.86 (0.97)
A*02:01	55 (10)	1.1	(0.52, 2.31)	0.8 (0.97)
A*02:03	96 (18)	0.88	(0.5, 1.54)	0.65 (0.94)
A*02:07	97 (18)	1.66	(0.92, 2.99)	0.09 (0.27)
A*11	263 (50)	1.43	(0.91, 2.24)	0.12 (0.29)
A*11:01	245 (47)	1.38	(0.88, 2.16)	0.17 (0.37)
A*24	163 (31)	1.26	(0.77, 2.05)	0.36 (0.69)
A*24:02	101 (19)	0.83	(0.47, 1.45)	0.51 (0.83)
**A*33:03**	**146 (28)**	**0.47**	**(0.29, 0.78)**	**0.003 (0.03)**
B*13	83 (16)	1.19	(0.63, 2.26)	0.59 (0.9)
B*13:01	70 (13)	1.44	(0.72, 2.88)	0.3 (0.63)
B*15	146 (28)	1.22	(0.74, 2.02)	0.44 (0.75)
B*15:02	83 (16)	1.11	(0.6, 2.07)	0.74 (0.97)
B*18	57 (11)	1.37	(0.68, 2.77)	0.38 (0.69)
B*40	104 (20)	1.63	(0.91, 2.9)	0.1 (0.27)
B*40:01	73 (14)	1.84	(0.94, 3.6)	0.08 (0.27)
B*46:01	139 (26)	1.67	(1, 2.78)	0.05 (0.24)
B*51	54 (10)	0.9	(0.42, 1.95)	0.79 (0.97)
**B*58:01**	**87 (17)**	**0.26**	**(0.14, 0.49)**	**2.53E-05 (0.0004)**
C*01	168 (32)	1.61	(0.99, 2.63)	0.06 (0.24)
C*01:02	168 (32)	1.61	(0.99, 2.63)	0.06 (0.24)
C*03	191 (36)	0.61	(0.38, 0.98)	0.04 (0.24)
**C*03:02**	**88 (17)**	**0.27**	**(0.14, 0.5)**	**2.72E-05 (0.0004)**
C*03:04	88 (17)	1.68	(0.91, 3.11)	0.1 (0.27)
C*04	95 (18)	1.06	(0.59, 1.88)	0.86 (0.97)
C*07	251 (48)	0.99	(0.63, 1.55)	0.97 (0.97)
C*07:02	159 (30)	0.98	(0.61, 1.6)	0.95 (0.97)
C*08	101 (19)	0.98	(0.56, 1.72)	0.95 (0.97)
C*08:01	100 (19)	0.98	(0.56, 1.72)	0.94 (0.97)

*q value was adjusted for multiple comparisons (29 HLA class I alleles). Age, sex, Fiebig stage, pre-ART VL, CD4 counts and ART regimen were adjusted in the model.
