# Supplementary material for: HLA-B*57 and B*58 Associate with Predictors of Reservoir Size in an Acutely Treated HIV Cohort
Source: AIDS Res Hum Retroviruses. 2023 Mar 3;39(3):114–8. doi: 10.1089/aid.2022.0082 (PMC9986004; doi:10.1089/aid.2022.0082)
Supplement: Supplemental data [file Suppl_TableS2.pdf]

Supplemental Table 2. Association of HLA alleles with Pre-ART VL (N = 526)

HLA Allele	N (%)	Beta	95% CI	P value (q value*)
A*02	253 (48)	-0.11	(-0.25, 0.03)	0.12 (0.63)
A*02:01	55 (10)	-0.12	(-0.35, 0.12)	0.32 (0.78)
A*02:03	96 (18)	-0.11	(-0.29, 0.08)	0.26 (0.74)
A*02:07	97 (18)	-0.01	(-0.19, 0.17)	0.93 (0.98)
A*11	263 (50)	0.17	(0.02, 0.31)	0.02 (0.27)
A*11:01	245 (47)	0.16	(0.02, 0.3)	0.03 (0.27)
A*24	163 (31)	-0.05	(-0.2, 0.11)	0.57 (0.98)
A*24:02	101 (19)	-0.002	(-0.18, 0.18)	0.98 (0.98)
A*33:03	146 (28)	-0.1	(-0.26, 0.06)	0.21 (0.74)
B*13	83 (16)	-0.02	(-0.22, 0.17)	0.82 (0.98)
B*13:01	70 (13)	-0.005	(-0.22, 0.21)	0.96 (0.98)
B*15	146 (28)	-0.18	(-0.34, -0.02)	0.03 (0.27)
B*15:02	83 (16)	-0.07	(-0.27, 0.12)	0.47 (0.98)
B*18	57 (11)	0.02	(-0.21, 0.25)	0.88 (0.98)
B*40	104 (20)	-0.01	(-0.19, 0.17)	0.9 (0.98)
B*40:01	73 (14)	-0.004	(-0.21, 0.2)	0.97 (0.98)
B*46:01	139 (26)	-0.02	(-0.19, 0.14)	0.77 (0.98)
B*51	54 (10)	0.18	(-0.05, 0.42)	0.13 (0.63)
B*58:01	87 (17)	-0.05	(-0.24, 0.14)	0.6 (0.98)
C*01	168 (32)	-0.02	(-0.17, 0.13)	0.81 (0.98)
C*01:02	168 (32)	-0.02	(-0.17, 0.13)	0.81 (0.98)
C*03	191 (36)	-0.03	(-0.18, 0.12)	0.7 (0.98)
C*03:02	88 (17)	-0.06	(-0.25, 0.13)	0.51 (0.98)
C*03:04	88 (17)	0.03	(-0.16, 0.22)	0.73 (0.98)
C*04	95 (18)	-0.16	(-0.34, 0.02)	0.09 (0.63)
C*07	251 (48)	0.09	(-0.06, 0.23)	0.24 (0.74)
C*07:02	159 (30)	0.04	(-0.11, 0.2)	0.6 (0.98)
C*08	101 (19)	-0.1	(-0.28, 0.08)	0.28 (0.74)
C*08:01	100 (19)	-0.12	(-0.3, 0.06)	0.19 (0.74)

*q value was adjusted for multiple comparisons (29 common HLA class I alleles). Age, sex, Fiebig stage and pre-ART CD4 counts were adjusted in each regression model.
