# Supplementary material for: HLA-B*57 and B*58 Associate with Predictors of Reservoir Size in an Acutely Treated HIV Cohort
Source: AIDS Res Hum Retroviruses. 2023 Mar 3;39(3):114–8. doi: 10.1089/aid.2022.0082 (PMC9986004; doi:10.1089/aid.2022.0082)
Supplement: Supplemental data [file Suppl_TableS3.pdf]

Supplementary Table 3. Association of HLA alleles with time to CD4 reconstitution after ART initiation (N = 526)

| HLA Allele | N (%) | OR | 95% CI | P value (q value*) |
|---|---|---|---|---|
| A*02 | 253 (48) | 1.22 | (0.79, 1.88) | 0.38 (0.67) |
| A*02:01 | 55 (10) | 0.61 | (0.32, 1.16) | 0.13 (0.67) |
| A*02:03 | 96 (18) | 1.46 | (0.8, 2.66) | 0.21 (0.67) |
| A*02:07 | 97 (18) | 1.25 | (0.71, 2.21) | 0.44 (0.68) |
| A*11 | 263 (50) | 0.76 | (0.49, 1.18) | 0.22 (0.67) |
| A*11:01 | 245 (47) | 0.68 | (0.43, 1.06) | 0.09 (0.67) |
| A*24 | 163 (31) | 1.24 | (0.76, 2.01) | 0.39 (0.67) |
| A*24:02 | 101 (19) | 1.43 | (0.79, 2.59) | 0.24 (0.67) |
| A*33:03 | 146 (28) | 1.17 | (0.72, 1.92) | 0.52 (0.71) |
| B*13 | 83 (16) | 1.07 | (0.58, 1.95) | 0.84 (0.9) |
| B*13:01 | 70 (13) | 1.43 | (0.72, 2.83) | 0.31 (0.67) |
| B*15 | 146 (28) | 1.01 | (0.62, 1.64) | 0.96 (0.96) |
| B*15:02 | 83 (16) | 0.8 | (0.45, 1.42) | 0.44 (0.68) |
| B*18 | 57 (11) | 1.66 | (0.76, 3.62) | 0.21 (0.67) |
| B*40 | 104 (20) | 1.03 | (0.6, 1.78) | 0.91 (0.95) |
| B*40:01 | 73 (14) | 0.9 | (0.49, 1.67) | 0.74 (0.82) |
| B*46:01 | 139 (26) | 1.55 | (0.92, 2.6) | 0.1 (0.67) |
| B*51 | 54 (10) | 1.18 | (0.55, 2.53) | 0.67 (0.8) |
| B*58:01 | 87 (17) | 1.36 | (0.74, 2.49) | 0.33 (0.67) |
| C*01 | 168 (32) | 1.44 | (0.89, 2.32) | 0.14 (0.67) |
| C*01:02 | 168 (32) | 1.44 | (0.89, 2.32) | 0.14 (0.67) |
| C*03 | 191 (36) | 1.26 | (0.79, 1.98) | 0.33 (0.67) |
| C*03:02 | 88 (17) | 1.36 | (0.74, 2.49) | 0.32 (0.67) |
| C*03:04 | 88 (17) | 1.18 | (0.65, 2.16) | 0.58 (0.74) |
| C*04 | 95 (18) | 0.79 | (0.46, 1.36) | 0.39 (0.67) |
| C*07 | 251 (48) | 0.92 | (0.59, 1.42) | 0.69 (0.8) |
| C*07:02 | 159 (30) | 0.86 | (0.54, 1.37) | 0.52 (0.71) |
| C*08 | 101 (19) | 0.79 | (0.46, 1.36) | 0.39 (0.67) |
| C*08:01 | 100 (19) | 0.84 | (0.49, 1.46) | 0.54 (0.71) |

*q value was adjusted for multiple comparisons (29 HLA class I alleles). Age, sex, Fiebig stage, pre-ART VL were adjusted in the model.
