# Supplementary material for: HLA-B*57 and B*58 Associate with Predictors of Reservoir Size in an Acutely Treated HIV Cohort
Source: AIDS Res Hum Retroviruses. 2023 Mar 3;39(3):114–8. doi: 10.1089/aid.2022.0082 (PMC9986004; doi:10.1089/aid.2022.0082)
Supplement: Supplemental data [file Suppl_TableS4.pdf]

Supplemental Table 4. Additional HLA analyses of low frequency (<5%) alleles (N = 526)

Outcome	HLA Allele	N (%)	OR	95% CI	P value
Time to VL suppression	B*57	11 (2)	0.07	(0.01, 0.64)	**0.02**
	B*35 (px)	9 (2)	0.56	(0.12, 2.52)	0.45

Covariates: age, sex, Fiebig stage, pre-ART VL, CD4 counts and ART regimen
