# Supplementary material for: HLA-B*57 and B*58 Associate with Predictors of Reservoir Size in an Acutely Treated HIV Cohort
Source: AIDS Res Hum Retroviruses. 2023 Mar 3;39(3):114–8. doi: 10.1089/aid.2022.0082 (PMC9986004; doi:10.1089/aid.2022.0082)
Supplement: Supplemental data [file Suppl_FigS1.pdf]

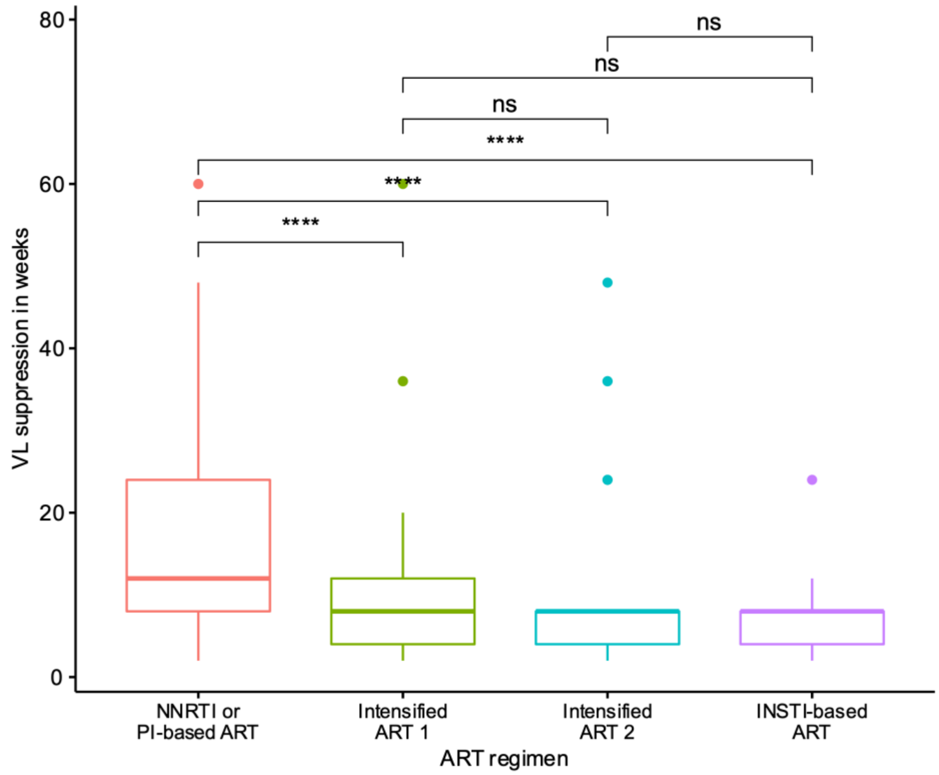

Supplementary Figure 1. Significant differences in VL suppression were identified based on the ART regimen. Non-nucleoside reverse transcriptase inhibitors (NNRTI), Protease Inhibitor (PI), Intensified ART 1 (efavirenz/maraviroc/raltegravir-based), Intensified ART 2 (dolutegravir/maraviroc- based), Integrase strand transfer Inhibitor (INSTI).
ns: not significant, **** $p < 0.0001$.
