# Supplementary material for: HLA-B*57 and B*58 Associate with Predictors of Reservoir Size in an Acutely Treated HIV Cohort
Source: AIDS Res Hum Retroviruses. 2023 Mar 3;39(3):114–8. doi: 10.1089/aid.2022.0082 (PMC9986004; doi:10.1089/aid.2022.0082)
Supplement: Supplemental data [file Suppl_FigS2.pdf]

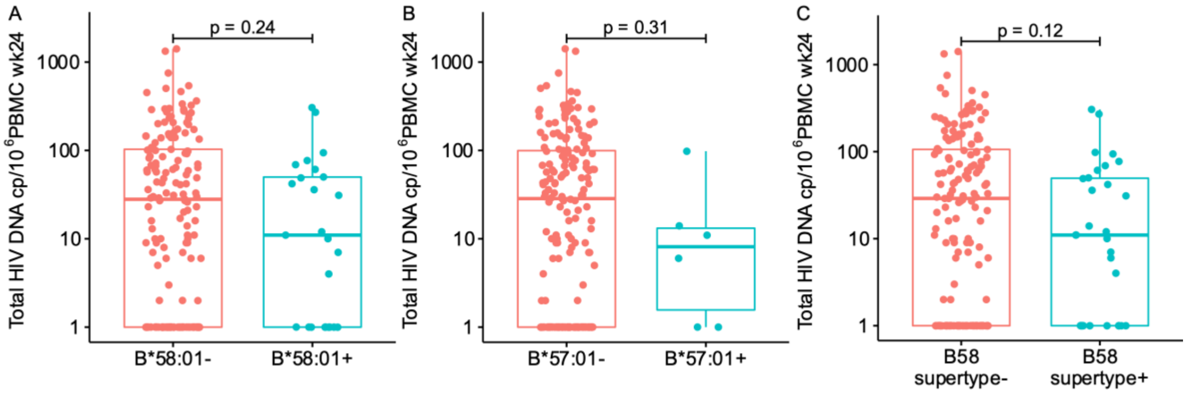

Supplementary Figure 2. No significant differences observed in HIV DNA levels in the presence of specific HLA alleles. Although not significant a trend towards statistical significance was observed when comparing presence of specific HLA alleles or combination with HIV reservoir size (N = 192). A) HLA-B*58 and B) HLA-B*57. C) HLA-B58 supertype. Statistical significance was determined by Mann-Whitney U test.
